# Interpretation of *BRCA2* Splicing Variants: A Case Series of Challenging Variant Interpretations and the Importance of Functional RNA Analysis

**DOI:** 10.1007/s10689-020-00224-y

**Published:** 2021-01-20

**Authors:** Paola Nix, Erin Mundt, Bradford Coffee, Elizabeth Goossen, Bryan M. Warf, Krystal Brown, Karla Bowles, Benjamin Roa

**Affiliations:** 1 Myriad Genetics, Inc., 320 Wakara Way, Salt Lake City, UT USA; 2 Third Wave Analytics, Inc., San Francisco, CA USA

**Keywords:** BRCA2, Hereditary cancer syndromes, Pathogenicity, RNA analysis, Splice variants

## Abstract

A substantial proportion of pathogenic variants associated with an increased risk of hereditary cancer are sequence variants affecting RNA splicing. The classification of these variants can be complex when both non-functional and functional transcripts are produced from the variant allele. We present four *BRCA2* splice site variants with complex variant interpretations (*BRCA2* c.68-3T>G, c.68-2A>G, c.425G>T, c.8331+2T>C). Evidence supporting a pathogenic classification is available for each variant, including in silico models, absence in population databases, and published functional data. However, comprehensive RNA analysis showed that some functional transcript may be produced by each variant. *BRCA2* c.68-3T>G results in a partial splice defect. For *BRCA2* c.68-2A>G and c.425G>T, aberrant splicing was shown to produce a potentially functional, in-frame transcript. *BRCA2* c.8331+2T>C may utilize a functional GC donor in place of the wild-type GT donor. The severity of cancer history for carriers of these variants was also assessed using a history weighting algorithm and was not consistent with pathogenic controls (carriers of known pathogenic variants in *BRCA2*). Due to the conflicting evidence, our laboratory classifies these *BRCA2* variants as variants of uncertain significance. This highlights the importance of evaluating new and existing evidence to ensure accurate variant classification and appropriate patient care.

**Supplementary information:**

The online version of this article (10.1007/s10689-020-00224-y) contains supplementary material, which is available to authorized users.

## Introduction

Pathogenic variants in *BRCA1* and *BRCA2* are associated with an increased risk of several cancers, including breast and ovarian cancer. Genetic testing is an important tool to identify individuals who may be at risk for developing cancer, for whom test results may have a considerable impact on clinical management. For example, carriers of a pathogenic variant in *BRCA1* or *BRCA2* are eligible for risk-reducing mastectomy and oophorectomy based on guidelines from the National Comprehensive Cancer Network (NCCN) [1]. Although the NCCN and other professional societies provide clear guidance for medical management based on the identification of pathogenic variants in cancer predisposition genes, the clinical significance of a variant is not always known at the time of testing.

The classification of some variants is evident based on a clear biological implication (i.e. protein truncation) that results in loss of function; however, that is not always the case. As a result, variant interpretation remains a challenge, even for genes as well-characterized as *BRCA1* and *BRCA2*. One class of variants that may be difficult to interpret are those with the potential to affect RNA splicing. A substantial fraction of disease-causing variants in cancer predisposition genes impact splicing [2]. The consequences of aberrant splicing can include skipping of one or more exons, activation of cryptic acceptor or donor sites, or intron retention. In many cases, this causes premature truncation or deletion of critical functional domains. Although classification of variants located within many canonical splice sites is often straightforward due to clear biological consequences, it is important that the impact on splicing of sequence variants outside the canonical sites be experimentally verified to ensure accurate variant classification.

A variety of computational tools have been developed to predict the effect a sequence variant may have on RNA splicing [3]. Although these methods often achieve a moderate degree of sensitivity and accuracy, the predicted splicing outcomes cannot be assumed to be accurate without experimental evidence. For example, in silico models cannot distinguish between a complete splice defect and a partial or “leaky” splice defect in which the variant allele produces some aberrant transcript and some normal transcript. Functional studies that directly assess splicing outcomes can provide useful data to aid variant classification [2, 4]. These may be performed by reverse transcription-polymerase chain reaction (RT-PCR) of RNA extracted from patient blood or lymphoblastoid cell lines (e.g. Montalban et al. [5]). Artificial minigene assays have been reported in cases where patient samples were unavailable (e.g. Fraile-Bethencourt et al. [6]). More recently, RNA sequencing methods have been applied to characterize and quantify mRNA transcripts [7–10].

The American College of Medical Genetics and Genomics and the Association for Molecular Pathology (ACMG/AMP) and Evidence-based Network for the Interpretation of Germline Mutant Alleles (ENIGMA) have provided guidelines for the interpretation of sequence variants, including variants that impact splicing [4, 11]. Guidelines include the types of evidence that can be used to classify a variant as pathogenic, along with their relative strengths for classification. The importance of functional analysis is emphasized for variants at a splice site, and several caveats are noted in relation to these variants. These include consideration of whether splicing variants lead to exon skipping that is in-frame and the potential generation of alternative transcripts [4, 11], both of which may result in the production of enough transcript to support normal protein function.

Here we describe four case examples of splice variants in *BRCA2* (*BRCA2* c.68-3T>G, c.68-2A>G, c.425G>T, and c.8331+2T>C) with complex variant interpretation, including new evidence from functional RNA analysis. In each case, the ACMG guidelines could support a pathogenic classification; however, additional data generated by our laboratory provides evidence that these variants may not be pathogenic.

## Materials and Methods

RNA analysis for variants predicted to impact splicing was performed using a research protocol approved by Quorum Review (now Advarra) independent review board (29678/1). Patients were eligible for this study if they were 18 or older at the time of hereditary cancer genetic testing (Myriad Genetic Laboratories), received testing as part of routine clinical care (i.e. not as part of a research study), and were found to carry a variant of interest. The health care provider was initially contacted regarding patient participation, followed by patient contact with a genetic counselor employed by the testing laboratory. Patients provided verbal informed consent before submitting a blood sample for RNA extraction.

Control blood samples were obtained from adult individuals who were not carriers of the variant of interest, had no history of cancer, and provided written informed consent. Control tissue samples utilized total RNA from human adult normal breast tissue purchased from BioChain Inc.

In addition to RNA analysis, other evidence relevant to variant classification was considered, including the results of a history weighting algorithm, in silico modeling, and relevant literature. We also evaluated the ClinVar classifications for each variant as of April 30, 2020 in order to compare our laboratory’s classification with other submitted classifications. The strength of the cited forms of evidence for pathogenicity was considered in light of the ACMG/AMP guidelines, which are utilized by clinical testing laboratories to guide variant classification in cancer predisposition genes.

### RNA Extraction and RT-PCR

Blood samples were collected in a Tempus Blood RNA tube (ThermoFisher Scientific) and total RNA was extracted using the Tempus Spin RNA Isolation kit (ThermoFisher Scientific) according to the manufacturer’s protocol. The extracted RNA was then quantified and stored at −80 °C. One to 2 μg of extracted total RNA was used to synthesize cDNA using the reverse transcriptase SuperScript IV VILO Master Mix with ezDNase enzyme (ThermoFisher Scientific) according to the manufacturer’s protocol. The cDNA was used as template in a standard PCR reaction with Takara Taq, hot start DNA polymerase (Takara Bio) for qualitative assessment by gel electrophoresis or by digital electrophoresis using the Agilent 2200 TapeStation system with D1000 ScreenTape (Agilent). Exon-specific primer sequences used for PCR amplification are shown in Table S1.

### Quantification of Allele-Specific Transcripts

Allele-specific sequence traces were obtained by single molecule PCR and Sanger sequencing. Template cDNA was diluted to the limit of detection to yield, on average, <1 template molecule per well in a multi-well plate. Primer pairs were combined into a 1 μM working stock and 2 μL of the stock was distributed to wells on a 384-well plate containing GoTaq HotStart DNA polymerase master mix (Promega). Template cDNA was diluted to 1–10 ng per reaction and distributed to wells. Each well was a separate PCR reaction that was used directly for automated Sanger sequencing. When possible, PCR primers were designed to flank the exon affected by the variant of interest, as well as a region containing a known heterozygous exonic single-nucleotide polymorphism (SNP). Each primer sequence included an M13 forward or reverse tag for use in Sanger sequencing.

The sequence traces from each individual reaction were then evaluated. In a given experiment, some wells failed to produce sequence indicating absence of template, some wells produced heterozygous traces indicating multiple templates, and some wells produced isolated sequence traces with allele-specific sequence. Only isolated traces were counted for the analysis. A minimum of 100 isolated sequence traces were analyzed for patient samples and a minimum of 50 isolated sequence traces from controls.

### History Weighting Algorithm

A history weighting-algorithm has been previously developed and validated by our laboratory for use in *BRCA2 *varriant classification [32]. In brief, the history weighting algorithm assumed that personal and family cancer history for individuals with a pathogenic variant will be more severe than for carriers of a benign variant in the same gene [32]. A score was assigned to a variant based on the personal and family history of gene-associated cancers among all eligible carriers of that variant. For *BRCA2*, relevant cancers included female breast cancer (including ductal carcinoma in situ), ovarian cancer, male breast cancer, pancreatic cancer, and prostate cancer.

The variant score was compared to scores for pathogenic and benign control variants in the same gene. The history weighting algorithm called the variant benign if the variant score was well within the benign control curve and did not significantly overlap with the pathogenic control curve (Figure S1a). The history weighting algorithm called the variant pathogenic if the variant score was well within the pathogenic control curve and did not significantly overlap with the benign control curve (Figure S1b). If the variant score had some significant overlap with both the benign and pathogenic control curves, no call was made. For *BRCA2*, the algorithm has been validated to have a positive predictive value of 99.71% and a negative predictive value of 99.90%.

## Results

### *BRCA2* c.68-3T>G

*BRCA2* c.68-3T>G is located three nucleotides upstream of exon 3, within the consensus splice acceptor site. Exon 3 encodes the PALB2 binding domain important for the interaction between PALB2 and BRCA2 and for proper localization of BRCA2 to sites of DNA damage [12]. A T>G sequence change at the −3 position is often associated with aberrant splicing, as pyrimidines are highly preferred at the −3 position of the intron [13]. Consistent with this, in silico models predicted that the variant would abolish splicing at the native acceptor (ACMG/AMP supporting evidence, Table 1). However, this information alone does not reach the threshold for a pathogenic classification, and the variant was classified as a variant of uncertain significance (VUS) in ClinVar by three submitting laboratories at the time of this analysis.

**Table 1:** Summary of evidence for variant classification. Items in bold represent evidence that the variant may not be pathogenic

*BRCA2* variant	Position^a^	In silico splice predictions^b,c^	Published literature	Population frequency^d^	Laboratory splice analysis	History weighting algorithm	Current laboratory classification
c.68-3T>G	Intron 2; consensus splice acceptor for exon 3	Abolish splicing at native acceptor (0.05>0); de novo cryptic acceptor (0>0.08); in-frame cryptic acceptor reduced (0.13 > 0.07)	None	16/244,208 chromosomes; 0.01% allele frequency	**Partial splice defect**; ∆3, ∆3-4, activation of *de novo* site, and normal transcript produced	**Benign**	VUS
c.68-2A>G	Intron 2; canonical splice acceptor for exon 3	Abolish splicing at native acceptor (0.05 > 0); in-frame cryptic acceptor 6nt into exon (0.13)	None	1/243,416 chromosomes; 0% allele frequency	**In-frame transcript produced**: activation of in-frame alternate acceptor; non-functional transcripts ∆3, ∆3-4	Inconclusive (trending benign)	VUS
c.425G>T	Last base of exon 4	Reduce strength of native donor (0.55 > 0.04)	Full splice defect; ∆4 [21]	Not present in large population databases	**In-frame transcripts produced**: ∆4-5, ∆4-7; non-functional transcripts produced: ∆4, ∆3-4, ∆3-5, ∆4-6	**Benign**	VUS
c.8331+2T>C	Intron 18; canonical splice donor for exon 18	Abolish splicing at native donor (0.74 > 0.03); in-frame cryptic donor 21nt into intron (0.1)	Complete ∆18 [24]; ∆18, ∆17q-18 [15] Partial splice defect ∆18, ∆17-18, ∆17q-18 [23]	Not present in large population databases	**Activation of GC splice donor**: ∆18, ∆17-18, ∆17q-18, and normal transcript produced	**Benign**	VUS

^a^Variants being located at the ±1 or 2 splice site in a gene where loss of function is a known mechanism of disease is considered very strong evidence of pathogenicity per ACMG/AMP guidelines [4]

^b^Laboratory-developed splice prediction tool based on Sheth et al. [33]; splice score ranges from 0 to 1

^c^In silico models that predict a deleterious effect are considered supporting evidence of pathogenicity per ACMG/AMP guidelines [4]

^d^The absence of a variant from large population databases (gnomAD) is considered moderate evidence that the variant may be pathogenic per ACMG/AMP guidelines [4]

#### RNA Analysis

**Fig. 1:**
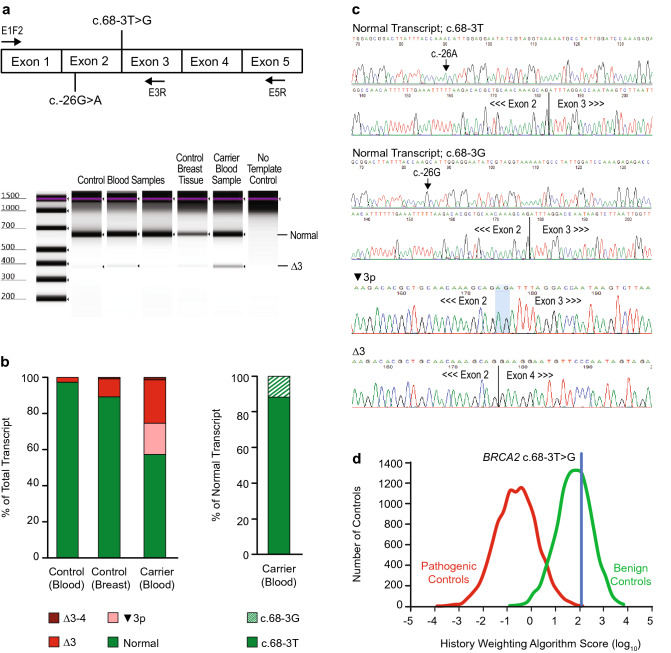
Analysis of *BRCA2* c.68-3T>G. (**a**) Schematic representation *BRCA2* regions amplified and digital electrophoresis of control and carrier samples amplified by E1F2-E5R. (**b**) Fraction of total transcript detected in control and carrier samples determined by quantification of transcripts from isolated traces. (**c**) Representative isolated sequence traces of observed transcripts from the variant carrier. The splice junction is indicated by a line. The 2 nucleotides inserted in the ▼3p transcript are shaded blue. (**d**) History weighting algorithm analysis based on 16 observations

The findings of RNA analysis for *BRCA2* c.68-3T>G are summarized in Fig. 1 and Table S2. Quantification of allele specific transcripts in a heterozygous carrier detected normal transcript, as well as three aberrant splice products: in-frame deletion of exon 3 (Δ3), out-of-frame deletion of exons 3–4 (Δ3–4), and utilization of the cryptic splice acceptor created by the variant (▼3p).

We analyzed 133 allele specific sequence traces and found that 57% (76/133) corresponded with normally spliced transcript, while 43% (57/133) were the result of aberrant splicing. Because c.68-3T>G is in the intron, and not part of the mRNA sequence, the informative variant c.-26G>A in the 5′ untranslated region was used as a proxy to distinguish transcripts produced by the variant allele (c.68-3G→c.-26G) from those produced by the wild-type allele (c.68-3T→c.-26A). Of the normal transcripts, 88% (67/76) were produced by the wild-type allele and 12% (9/76) by the variant allele. Similar ratios were observed when specifically amplifying the normal transcript with a reverse primer placed within exon 3, where 84% (61/73) of transcripts were from the wild-type allele and 16% (12/73) from the variant allele. Taken together, these data show that c.68-3T>G causes a partial splice defect.

Both wild-type and variant alleles also contributed to the aberrant transcripts. The patient’s wild-type allele produced 5% (3/57) of the aberrant transcripts, including Δ3 and Δ3–4. This result is not unexpected, as extensive alternative splicing is known to naturally occur in this region of *BRCA2* [19, 20]. The remaining 95% of aberrant transcripts were produced by the variant allele and included Δ3 (53%, 30/57), Δ3–4 (2%, 1/57), and ▼3p (40%, 23/57), all of which are expected to be deleterious to protein function.

#### Variant Classification

In combination with in silico models, the identification of aberrant splicing could be considered sufficient for a pathogenic classification of c.68-3T>G. However, comprehensive RNA analysis revealed that c.68-3T>G results in a partial splicing defect, with some functional transcript produced by the c.68-3G variant allele. Additional clinical information was provided by the history weighting algorithm, which called this variant benign based on the relative severity of personal and family cancer history for c.68-3T>G carriers compared to negative controls (individuals with known benign variants in *BRCA2*) and positive controls (individuals with known pathogenic variants in *BRCA2*; Fig. 1d). This indicates that the approximately 15% of normal transcript produced by the variant allele may be sufficient to support normal BRCA2 function. Considering this conflicting evidence, our laboratory classifies *BRCA2* c.68-3T>G as VUS (Table 1).

### *BRCA2* c.68-2A>G

*BRCA2* c.68-2A>G is located two nucleotides upstream of exon 3, within the canonical splice acceptor site. The sequence change affects the invariant AG dinucleotide of the splice acceptor and is predicted to abolish usage of this splice site by in silico methods. At the time of this analysis, the variant was classified as likely pathogenic in ClinVar by three submitting laboratories. The evidence for classification included the variant position at the −2 nucleotide of the intron (ACMG/AMP very strong evidence) and in silico models (ACMG/AMP supporting evidence; Table 1).

Upon initial observation of this variant, our laboratory classified c.68-2A>G as VUS due to the presence of an in-frame cryptic acceptor six nucleotides into the exon. In silico predictions and manual review found that this cryptic splice acceptor scored better than the normal splice acceptor site (Table 1). Utilization of the cryptic splice site would produce a transcript with the in-frame deletion of two amino acids (p.Asp23_Leu24del). Although these amino acids are conserved within the PALB2 binding peptide, they are not part of the core binding motif that includes Trp31, Phe32 and Leu35, and structural modeling of the deleted amino acids is equivocal [14]. Therefore, the functional consequences of this deletion were considered uncertain.

#### RNA Analysis

**Fig. 2:**
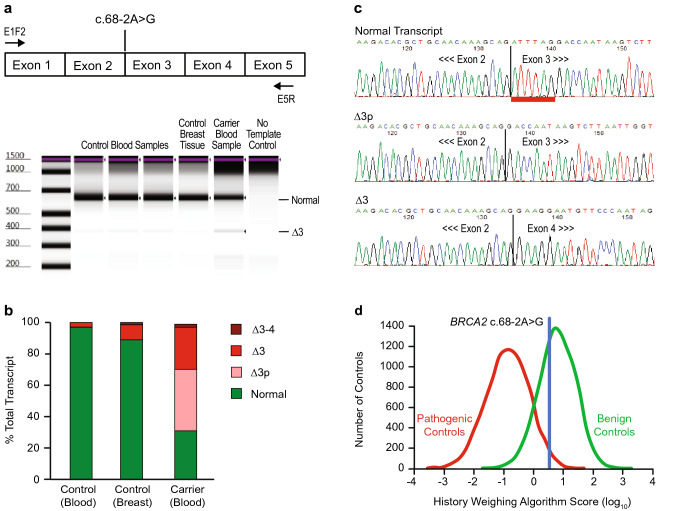
Analysis of *BRCA2* c.68-2A>G. (**a**) Schematic representation *BRCA2* regions amplified and digital electrophoresis of control and carrier samples. (**b**) Fraction of total transcript detected in control and carrier samples determined by quantification of transcripts from isolated traces. (**c**) Representative isolated sequence traces of observed transcripts from the variant carrier. The splice junction is indicated by a line. The 6 nucleotides missing from Δ3p are underlined in the normal transcript trace. (**d**) History weighting algorithm analysis based on 11 observations

The findings of RNA analysis for *BRCA2* c.68-2A>G are summarized in Fig. 2 and Table S3. Quantification of transcripts in a heterozygous carrier yielded normal transcript as well as three aberrantly spliced transcripts. We analyzed 135 isolated sequence traces and found that 31% (42/135) corresponded with normal transcript. The remaining 69% (93/135) of transcripts were the result of aberrant splicing: 39% (53/135) utilized the in-frame cryptic donor 6 nucleotides into the exon (Δ3p), 27% (37/135) skipped exon 3 (Δ3), and 2% (3/135) skipped exons 3–4 (Δ3–4). A heterozygous SNP was not included in this analysis; however, a partial splice defect is unlikely due to the loss of the canonical AG.

#### Variant Classification

RNA analysis showed the production of the non-functional Δ3 and Δ3–4 splice isoforms. Use of the cryptic acceptor site 6 nucleotides into the exon was not observed in normal controls, nor has it been described in published studies assessing naturally occurring splice isoforms of *BRCA2* [19, 20]. Based on these data, the possibility remains that a significant portion of transcript produced by the variant c.68-2G allele may be functional due to utilization of the in-frame cryptic splice acceptor. *BRCA2* c.68-2A>G did not meet the threshold to be classified as benign by the history weighting algorithm, though it was trending in that direction after 11 observations of the variant (Fig. 2d). As a result, our laboratory continues to classify *BRCA2* c.68-2A>G as VUS.

### *BRCA2* c.425G>T

*BRCA2* c.425G>T is a missense change located at the last base of exon 4, making it part of the consensus splice donor site. Variation at this position may cause aberrant splicing due to a decrease in the base pairing interactions between the U1 snRNP and the primary transcript. Consequently, in silico methods predicted that c.425G>T will significantly impact splicing (Table 1).

*BRCA2* c.425G>T was classified as likely pathogenic in ClinVar by six submitting laboratories at the time of this analysis. The cited evidence included the variant position at the last nucleotide of the exon (ACMG/AMP moderate evidence), in silico models (ACMG/AMP supporting evidence), absence of the variant from large population databases (ACMG/AMP moderate evidence), and published RNA analysis showing skipping of exon 4 with predicted protein truncation (ACMG/AMP strong evidence) (Table 1). Brandao et al. demonstrated complete exon 4 skipping in a patient sample with no contribution of the variant allele to the normal transcript [21]. Based on this body of evidence, this variant was also classified as pathogenic by our laboratory.

#### RNA Analysis

**Fig. 3:**
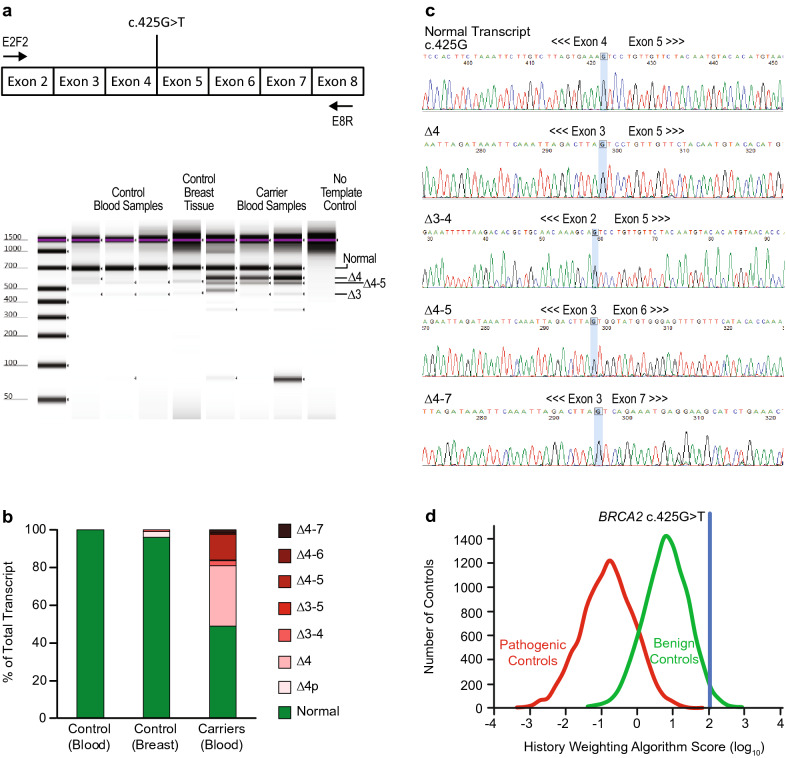
Analysis of *BRCA2* c.425G>T. (**a**) Schematic representation *BRCA2* regions amplified and digital electrophoresis of control and carrier samples. Some expected products may be obscured by the presence of similarly sized bands and/or may be present at too low a concentration to be visualized by electrophoresis. Not all individual bands are labeled, as the bands were not purified and sequenced separately. (**b**) Fraction of total transcript detected in control and carrier samples determined by quantification of transcripts from isolated traces. (**c**) Representative isolated sequence traces of observed transcripts from the variant carrier. The last base of the exon is highlighted blue. (**d**) History weighting algorithm analysis based on 10 observations

The findings of RNA analysis for *BRCA2* c.425G>T are summarized in Fig. 3 and Table S4. Quantification of RNA transcripts from three related individuals yielded normal transcript as well as multiple aberrantly spliced products. Similar results were observed in each of the variant carriers, with a combined total of 449 isolated sequence traces analyzed. Overall, 49% of traces (220/449) were from normal transcripts, which were produced exclusively by the wild-type c.425G allele. This indicates that the variant allele only produces abnormally spliced mRNA. The remaining 51% of traces (229/449) showed abnormally spliced transcripts. Of the aberrantly spliced transcripts specifically affecting exon 4, a majority (62%, 143/229) showed skipping of exon 4 (Δ4), which is expected to be pathogenic. The next most abundant product was Δ4–5 with 27% of traces (62/229), followed by Δ3–4 (6%, 13/229), Δ4–7 (3%, 7/229), Δ4–6 (1%, 3/229), and finally, Δ3–5 (<1%, 1/229).

As stated previously, the region of *BRCA2* including exons 3–7 is known to naturally undergo extensive alternative splicing [19, 20]. One of the most frequent alternative splice products is skipping of exon 5 (Δ5), which is present in 2–3% of total *BRCA2* transcript [19]. As a single exon-skipping event, ∆5 is predicted to result in frameshift and premature truncation. However, in the patient samples analyzed here, skipping of exon 4 caused by the c.425G>T variant in combination with skipping of exon 5 (i.e. Δ4–5) restored the reading frame. Similarly, Δ4–7 maintained the reading frame and was observed in patient samples.

#### Variant Classification

In total, 30% of the aberrant transcripts (69/229 traces) produced by the variant c.425T allele remain in frame. These in-frame isoforms delete amino acids that are not known to be critical for BRCA2 function. Studies have shown that a transcript lacking exons 4–7 retains function since it rescues the lethality of BRCA2 null cells, shows normal sensitivity to DNA damaging agents, exhibits normal DNA repair activity, and renders engineered mice viable and fertile [16, 22]. This raises the possibility that in-frame, alternative mRNA isoforms associated with *BRCA2* c.425G>T (i.e. in-frame Δ4–5) may also retain function. Additional clinical evidence was provided by the history weighting algorithm, which called the *BRCA2* c.425G>T variant benign (Fig. 3d). Taking this new evidence into consideration, our laboratory reclassified *BRCA2* c.425G>T to VUS. The ENIGMA consortium also recommends a VUS classification for this variant based on a posterior probability of pathogenicity of 0.92, which does not meet their threshold of 0.95 for a likely pathogenic classification [17].

### *BRCA2* c.8331+2T>C

*BRCA2* c.8331+2T>C is located two nucleotides downstream of exon 18, which affects the canonical GT dinucleotide of the splice donor site and is predicted to abolish splicing by in silico methods (Table 1). This variant was classified as pathogenic or likely pathogenic in ClinVar by six submitting laboratories at the time of this analysis. The evidence for classification included the variant position at the +2 nucleotide of the intron (ACMG/AMP very strong evidence), in silico models (ACMG/AMP supporting evidence), absence of the variant from large population sequence databases (ACMG/AMP moderate evidence), and published RNA analysis showing skipping of exon 18 along with other aberrant transcripts that are predicted to cause protein truncation (ACMG/AMP strong evidence) (Table 1) [15, 23, 24]. Based on this evidence, our laboratory initially classified this variant as likely pathogenic.

#### RNA Analysis

**Fig. 4:**
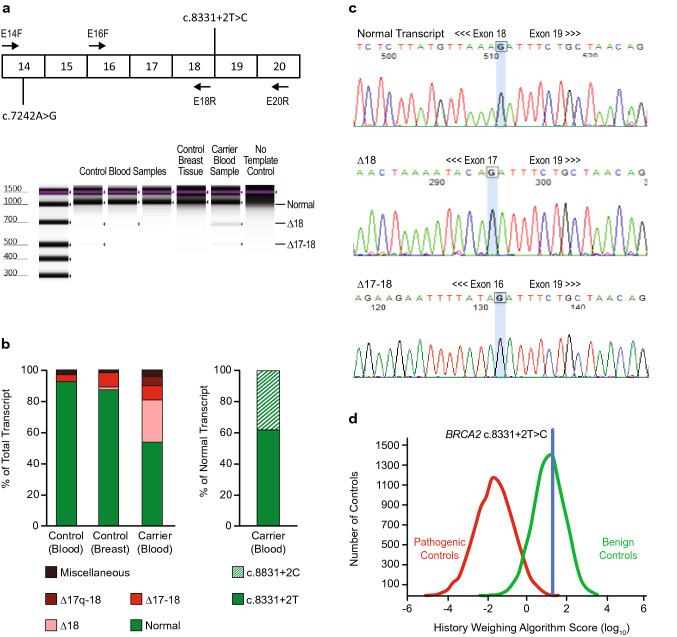
Analysis of *BRCA2* c.8331+2T>C. (**a**) Schematic representation *BRCA2* regions amplified and digital electrophoresis of control and carrier samples amplified by E16F-E20R. (**b**) Fraction of total transcript detected in control and carrier samples determined by quantification of transcripts from isolated traces. (**c**) Representative isolated sequence traces of observed transcripts from the variant carrier. The last base of the exon is highlighted blue. (**d**) History weighting algorithm analysis based on 18 observations

The findings of RNA analysis for *BRCA2* c.8331+2T>C are summarized in Fig. 4 and Table S5. Quantification of RNA transcripts in a heterozygous carrier yielded normal transcript as well as three aberrantly spliced transcripts. We analyzed 119 isolated sequence traces and found that 54% (64/119) were from normal transcript and 46% (55/119) from aberrantly spliced transcripts. A majority (58%, 32/55) of aberrantly spliced transcripts showed skipping of exon 18 (Δ18), which is expected to be pathogenic. Other aberrant transcripts included Δ17–18 (20%, 11/55) and Δ17q-18 (13%, 7/55), which resulted from use of a cryptic donor site within exon 17 causing the deletion of 151 nucleotides of exon 17 as well as deletion of exon 18. In a small number of traces (9%, 5/55), we observed miscellaneous transcripts using a non-canonical cryptic donor within exon 18 spliced to downstream intronic or exonic sequences.

Additional single molecule PCR was performed to specifically amplify the normal transcript encoding exon 18 by pairing a reverse primer within exon 18 with a forward primer in exon 14 that encodes the informative heterozygous variant c.7242A>G (wild-type c.8331+2T → c.7242A; variant c.8331+2C → c.7242G). This allowed for allele-specific quantification of the normal transcript where we observed that 62% (62/100) of traces were produced by the wild-type allele and 38% (38/100) by the variant allele. These data suggest that the variant c.8331+2C allele contributes to a significant fraction of the normal transcript, in addition to causing a splicing defect resulting in the out-of-frame skipping of exon 18 or exons 17–18.

#### Variant Classification

RNA analysis demonstrated that the splice defect associated with *BRCA2* c.8331+2T>C produced a significant amount of aberrant splicing involving skipping of exon 18. Each of the alternative transcripts observed are predicted to result in a frameshift with premature truncation. However, examination of normal transcript encoding exon 18 showed that both the c.8331+2T wild-type allele and c.8331+2C variant allele produced normal transcript. These data demonstrate that the GC donor created by the variant is likely utilized and may produce sufficient levels of *BRCA2* transcript for normal protein function. Additional evidence is provided by the history weighting algorithm, which provided a benign call for the c.8331+2T>C variant (Fig. 4d). Based on the collective evidence, *BRCA2* c.8331+2T>C was reclassified to VUS. The ENIGMA consortium also recommends a classification of VUS based on a posterior probability of pathogenicity of 0.86 [17].

## Discussion

Here we present four *BRCA2* variants predicted to impact RNA splicing. In each case, ACMG/AMP guidelines for variant interpretation could support a pathogenic or likely pathogenic classification. However, the interpretation of some splice site variants may be complicated if the variant allele produces transcript resulting in functional protein. This was the case for the variants examined, where some functional transcript was produced due to a partial splice defect, a functional in-frame deletion, or utilization of a GC donor site.

Overall, it is relatively uncommon for pathogenic variants to be downgraded based on a re-evaluation of the available evidence. For example, Mersch et al. showed that only 0.7% of variants in cancer predisposition genes initially classified as pathogenic by our laboratory were downgraded over a 10-year period [25]. Although uncommon, it is critical that new and existing evidence be carefully considered by testing laboratories in order to provide the most accurate variant classification and avoid unnecessary medical interventions. For the *BRCA2* case examples presented here, our RNA analysis and history weighting algorithm provided contradictory evidence to the existing evidence that initially supported a pathogenic classification. As a result, the body of evidence now available for these variants is inconclusive and medical management for individuals carrying one of these variants should be based on personal and family history. Additional strong, clinical evidence, such as an in trans observation in a patient without features of Fanconi anemia [4], would be needed to rule out the possibility of increased cancer risk in order to reach a benign classification.

### Variants Causing Incomplete Splice Defects May not be Pathogenic

The interpretation of variants with a partial splice defect can be especially problematic in variant classification. These variants produce aberrant transcripts with proven deleterious effects as well as normal transcripts. Importantly, it is not always possible to determine if the amount of normal transcript is sufficient for normal biological function from RNA analysis alone. Classification of variants with partial splice defects should be approached with caution in the absence of other strong supporting clinical evidence.

For *BRCA2* c.68-3T>G, evidence from in silico models and published case studies are supportive of a pathogenic classification in accordance with ACMG/AMP guidelines. RNA analysis identifying a significant fraction of aberrant splice products with known pathogenic effects would meet the level of evidence considered sufficient for a likely pathogenic classification by many testing laboratories [8]. However, it is important that RNA analysis be thorough when it is the primary line of evidence in clinical variant classification. In such cases, allele-specific transcripts should be fully quantified to rule out the possibility of a partial defect. This is especially relevant for intronic splice variants, which frequently cause partial splice defects but are not present in the coding sequence. In such cases, it is not possible to fully quantify the splice defect and determine if the variant allele also produces normal transcript without an informative exonic variant.

In the case of *BRCA2* c.68-3T>G, the history weighting algorithm call of benign suggests that the expression of normal transcript from the variant allele may be sufficient for BRCA2 function. Similar observations were made following RNA analysis of *BRCA2* c.68-5A>G by Gelli et al. and of *BRCA2* c.68-7T>A by Colombo et al. [23, 26]. These studies demonstrated partial splice defects for these variants and the authors suggested that additional evidence is needed before concluding pathogenicity. More recently, Tubeuf et al. estimated the threshold for transcript expression from a variant allele using BRCA2 exon 3 as a model [18]. Combined data from RNA analysis and functional studies in a mouse embryonic stem cell assay indicate that BRCA2 may be particularly tolerant to decreases in transcript expression. Based on evidence from variants in exon 3, Tubeuf et al. suggest a conservative threshold of 4% expression of the normal transcript from the variant allele and therefore any variant producing >4% would be considered VUS in the absence of additional supporting evidence [18].

### Aberrant Splicing May Produce Functional, In-Frame Transcripts

*BRCA2* c.425G>T was shown by our analysis and by others to cause a full splice defect, with no normal full-length transcript produced by the variant allele [21]. However, the in-frame ∆4–5 isoform affects a portion of *BRCA2* with no known function and restored the reading frame in about 30% of transcripts produced by the variant allele. We conclude that this deletion may be functional based on previous biochemical analysis showing that the larger in-frame ∆4–7 deletion rescues the lethality of BRCA2 null cells [22]. Furthermore, Mesman et al. observed that cells expressing c.425G>T retain 66% activity in a homology directed repair assay [27].

De la Hoya et al. proposed a similar rescue mechanism for *BRCA1* c.594-2A>C based on the production of the in-frame Δ9–10 isoform, which retains the functional domains of BRCA1 [28]. The authors infer that *BRCA1* can tolerate a substantial reduction in the amount of normal (i.e. full-length) transcript and still produce enough functional protein to support normal biological function. Specifically, the authors propose that any allele permitting 20–30% expression of functional BRCA1 should not be considered pathogenic [28]. This rescue mechanism for *BRCA1* draws a striking parallel to the findings from Tubeuf et al., which shows tolerance to a substantial decrease in normal *BRCA2* transcript [18].

The history weighting algorithm call of benign for *BRCA2* c.425G>T suggests that a variant allele producing as little as about 10% of functional transcript may not confer a high cancer risk. These data also have implications for other variants at the canonical +1/+2 donor sequence that would similarly abolish splicing, causing skipping of exon 4 with potential rescue by the Δ4–5 transcript. The possibility of rescue by alternate in-frame transcripts additionally highlights the need for awareness of the full range of naturally occurring splice isoforms for a given gene, as this will inform primer design. It may not be appropriate to design primers immediately flanking the exon of interest if those exons are subject to alternative splicing.

*BRCA2* c.68-2A>G also showed potential rescue by an in-frame, aberrant transcript. In this case, nearly 60% of aberrant transcripts resulted from use of the in-frame cryptic splice site. Activation of this alternate acceptor results in the deletion of two amino acids (Asp23 and Leu24). Although the consequence of this deletion on protein function is uncertain, our results raise the possibility that a functional transcript could be produced. Tubeuf et al. also showed activation of the in-frame cryptic acceptor in a similar variant at this junction, c.68-1G>A [18]. Therefore, we believe that the classification of any −1/−2 variant at the *BRCA2* exon 3 splice acceptor should be a VUS in the absence of additional strong evidence of pathogenicity.

### GT>GC Variants at the Consensus Donor May be Functional

The vast majority of splice donor sites encode GT at the first and second nucleotide of the intron sequence [29]. Sequence changes to the +1/+2 position are often considered likely pathogenic without confirmation from in vitro studies [4, 30]. However, about 1% of human introns encode a noncanonical GC at the splice donor site, implying that GC donors can be recognized and spliced appropriately in some cases [29].

Our analysis of *BRCA2* c.8331+2T>C identified that the variant allele produced nearly 40% of the normal transcript. Gelli et al. also described splicing associated with c.8331+2T>C and observed biallelic expression of the normal transcript [23]. These data demonstrate that the GC donor created by the variant is likely utilized and may produce sufficient levels of *BRCA2* transcript for normal protein function. Lin et al. recently estimated that 15–18% of GT>GC donors are capable of generating wild-type transcripts, with wild-type expression levels between 1% and 84% [31]. These data indicate that functional GT>GC donors are not uncommon and are not invariably pathogenic. However, it is not possible to predict which GT>GC change will generate a transcript expressed at adequate levels without experimental verification. With the addition of the history weighting algorithm call of benign, we agree with the conclusion from Gelli et al. that *BRCA2* c.8331+2T>C be classified VUS.

## Conclusion

ACMG/AMP guidelines provide direction on the types and strength of evidence supporting pathogenicity for variants predicted to affect RNA splicing [4]. However, these examples, as well as others cited in the literature, have underscored the need for caution in applying these guidelines to splice variant interpretation. In some cases, a variant that produces an aberrant transcript may also produce enough transcript to support normal protein function. As illustrated here, this may arise as part of a partial splice defect, an in-frame deletion, or utilization of a functional GC donor site. Thorough RNA analysis is an important tool to identify whether these scenarios are possible for an individual variant. The use of a history weighting algorithm to assess the severity of personal and family cancer history also provided supporting information for the four variants presented here. In the case of *BRCA2* c.425G>T and c.8331+2T>C, a benign call from the history weighting algorithm highlighted the need for additional RNA analyses despite the evidence that those variants were pathogenic based on ACMG/AMP guidelines. The availability of new evidence will sometimes result in a meaningful change in variant classification that may impact medical management decisions. As such, clinical testing laboratories must continue to evaluate all existing and new evidence to ensure accurate variant classifications to support appropriate medical management.

## Supplementary information

Fig. S1 History weighting algorithm graphs illustrating (a) a benign call for *BRCA2* c.68-7T>A and (b) a pathogenic call for *BRCA2* c.316+5G>A. The variant-specific score (blue line) was compared to pathogenic composite control variants (red curve) and benign composite control variants (green curve). The log of the score is plotted on the x-axis with the number of control variants plotted on the y-axis (DOCX 98 kb)

Table S1 Exon-specific primer sequences used for PCR amplification (DOCX 13 kb)

Table S2 Quantification of transcripts produced by *BRCA2* c.68-3T>G and controls (DOCX 14 kb)

Table S3 Quantification of transcripts produced by *BRCA2* c.68-2T>G and controls (DOCX 13 kb)

Table S4 Quantification of transcripts produced by *BRCA2* c.425G>T and controls (DOCX 14 kb)

Table S5 Quantification of transcripts produced by *BRCA2* c.8331+2T>C and controls (DOCX 13 kb)

## Data Availability

The data that supports the findings of this study are available in text.
