# Supplementary figures and images for: Interpretation of BRCA2 Splicing Variants: A Case Series of Challenging Variant Interpretations and the Importance of Functional RNA Analysis
Source: Fam Cancer. 2021 Jan 20;21(1):7–19. doi: 10.1007/s10689-020-00224-y (PMC8799590; doi:10.1007/s10689-020-00224-y)

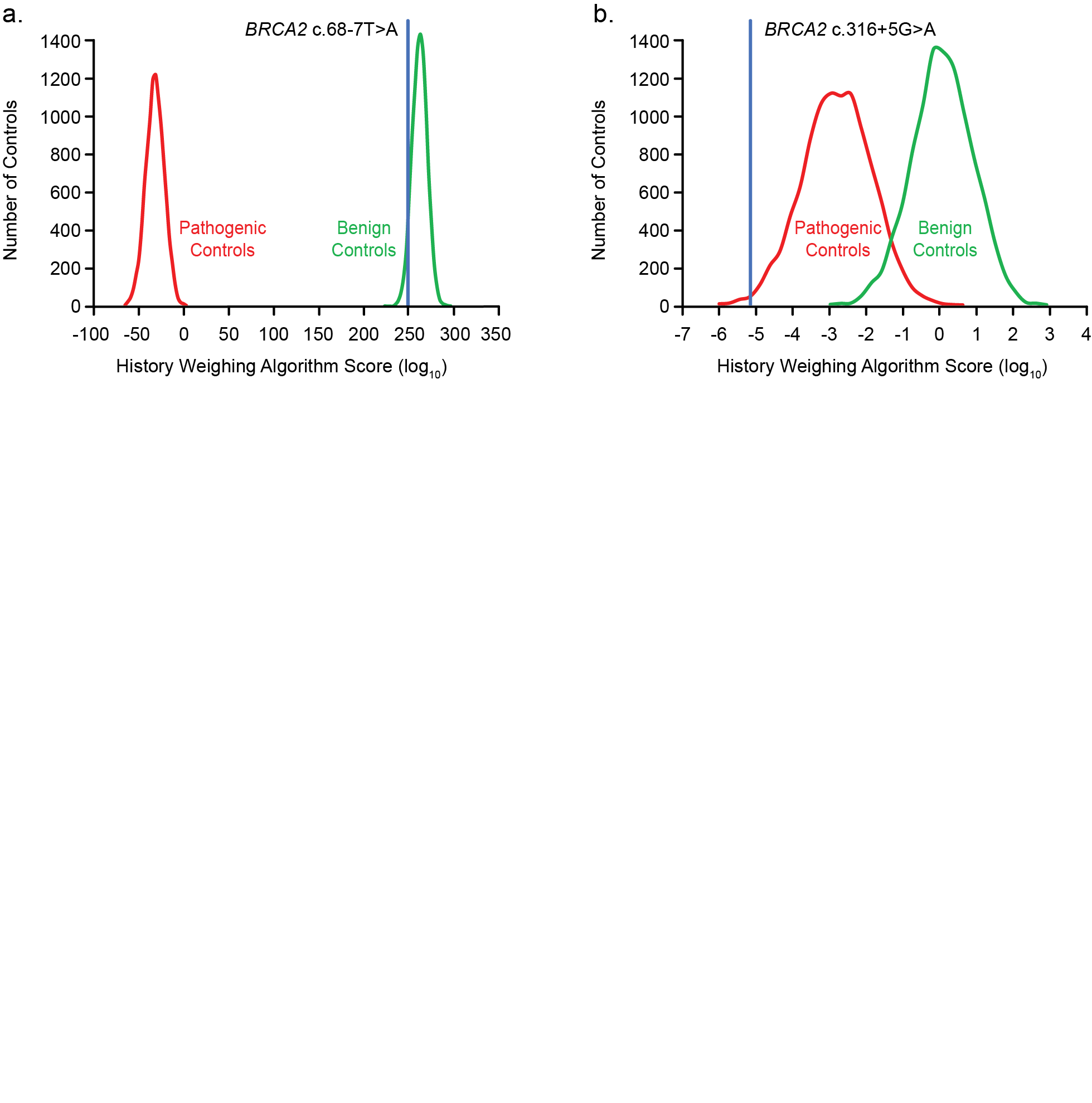

Supplement: Supplementary file 1 — History weighting algorithm graphs illustrating (a) a benign call for BRCA2 c.68-7T>A and (b) a pathogenic call for BRCA2 c.316+5G>A. The variant-specific score (blue line) was compared to pathogenic composite control variants (red curve) and benign composite control variants (green curve). The log of the score is plotted on the x-axis with the number of control variants plotted on the y-axis (DOCX 98 kb) [file 10689_2020_224_MOESM1_ESM.docx]
