# Supplementary material for: Interpretation of BRCA2 Splicing Variants: A Case Series of Challenging Variant Interpretations and the Importance of Functional RNA Analysis
Source: Fam Cancer. 2021 Jan 20;21(1):7–19. doi: 10.1007/s10689-020-00224-y (PMC8799590; doi:10.1007/s10689-020-00224-y)
Supplement: Supplementary file 2 — Exon-specific primer sequences used for PCR amplification (DOCX 13 kb) [file 10689_2020_224_MOESM2_ESM.docx]

| **Name** | **M13 Tail** | **Gene-specific Sequence** | **5'-3' Primer sequence** |
| --- | --- | --- | --- |
| E1F2 | GTTTTCCCAGTCACGACG | GAGGGGACAGATTTGTGACC | GTTTTCCCAGTCACGACGGAGGGGACAGATTTGTGACC |
| E2F2 | GTTTTCCCAGTCACGACG | CTATTGGATCCAAAGAGAGGC | GTTTTCCCAGTCACGACGCTATTGGATCCAAAGAGAGGC |
| E3R | AGGAAACAGCTATGACCAT | GAGTCAGCCCTTGCTCTTTG | AGGAAACAGCTATGACCATGAGTCAGCCCTTGCTCTTTG |
| E5R1 | AGGAAACAGCTATGACCAT | CATGTGTACATTGTAGAACAACAGG | AGGAAACAGCTATGACCATCATGTGTACATTGTAGAACAACAGG |
| E8R | AGGAAACAGCTATGACCAT | GGAAATACAGTTTCAGATGCTTC | AGGAAACAGCTATGACCATGGAAATACAGTTTCAGATGCTTC |
| E14F | GTTTTCCCAGTCACGACG | CGCACCTGGTCAAGAATTTC | GTTTTCCCAGTCACGACGCGCACCTGGTCAAGAATTTC |
| E16F | GTTTTCCCAGTCACGACG | GCAGAGTCTTTTCAGTTTCACAC | GTTTTCCCAGTCACGACGGCAGAGTCTTTTCAGTTTCACAC |
| E18R | AGGAAACAGCTATGACCAT | GCCGATCTTCTGCTTCTATC | AGGAAACAGCTATGACCATGCCGATCTTCTGCTTCTATC |
| E20R2 | AGGAAACAGCTATGACCAT | CATGTTCTTCAAATTCCTCCTG | AGGAAACAGCTATGACCATCATGTTCTTCAAATTCCTCCTG |
