# Supplementary material for: Interpretation of BRCA2 Splicing Variants: A Case Series of Challenging Variant Interpretations and the Importance of Functional RNA Analysis
Source: Fam Cancer. 2021 Jan 20;21(1):7–19. doi: 10.1007/s10689-020-00224-y (PMC8799590; doi:10.1007/s10689-020-00224-y)
Supplement: Supplementary file 3 — Quantification of transcripts produced by BRCA2 c.68-3T>G and controls (DOCX 14 kb) [file 10689_2020_224_MOESM3_ESM.docx]

| **Region Amplified** | **Transcript Observed** | **Control Blood^†^** | **Control Breast Tissue^†^** | **Variant Carrier^‡^** | **cDNA** | **Protein** |
| --- | --- | --- | --- | --- | --- | --- |
| Exons 1-5 | Full-length | 97% (200/206) | 89% (174/195) | c.-26A: 50% (67/133)  c.-26G: 7% (9/133) |  |  |
|  | ▼3p | 0 | 0 | c.-26A: 0%  c.-26G: 17% (23/133) | c.66_67dup | p.Asp23Glufs*3 |
|  | ∆3 | 3%  (6/206) | 10% (19/195) | c.-26A: 2% (2/133)  c.-26G: 23% (30/133) | c.68_316del | p.Asp23_Leu105del |
|  | ∆3-4 | 0 | 1%  (2/195) | c.-26A: <1% (1/133)  c.-26G: <1% (1/133) | c.68_425del | p.Asp23Valfs*10 |
| Exons 1-3 | Full-length | 100% (44/44) | 100% (51/51) | c.-26A: 47% (61/133)  c.-26G: 9% (12/133) |  |  |
|  | ▼3p | 0 | 0 | c.-26A: 47% (61/131)  c.-26G: 9% (12/131) | c.66_67dup | p.Asp23Glufs*3 |

† % (n isolated traces/total traces)

‡ Allele of origin: % (n isolated traces/total traces)
