# Supplementary material for: Interpretation of BRCA2 Splicing Variants: A Case Series of Challenging Variant Interpretations and the Importance of Functional RNA Analysis
Source: Fam Cancer. 2021 Jan 20;21(1):7–19. doi: 10.1007/s10689-020-00224-y (PMC8799590; doi:10.1007/s10689-020-00224-y)
Supplement: Supplementary file 4 — Quantification of transcripts produced by BRCA2 c.68-2T>G and controls (DOCX 13 kb) [file 10689_2020_224_MOESM4_ESM.docx]

| **Region Amplified** | **Transcript Observed** | **Control Blood^†^** | **Control Breast Tissue^†^** | **Variant Carrier^†^** | **cDNA** | **Protein** |
| --- | --- | --- | --- | --- | --- | --- |
| Exons 1-5 | Full-length | 97% (200/206) | 89% (174/195) | 31% (42/135) |  |  |
|  | Δ3p | 0 | 0 | 39% (53/135) | c.68_73del | p.Asp23_Leu24del |
|  | ∆3 | 3%  (6/206) | 10% (19/195) | 27% (37/135) | c.68_316del | p.Asp23_Leu105del |
|  | ∆3-4 | 0 | 1% (2/195) | 2% (3/135) | c.68_425del | p.Asp23_Valfs*10 |

† % (n isolated traces/total traces)
