# Supplementary material for: Interpretation of BRCA2 Splicing Variants: A Case Series of Challenging Variant Interpretations and the Importance of Functional RNA Analysis
Source: Fam Cancer. 2021 Jan 20;21(1):7–19. doi: 10.1007/s10689-020-00224-y (PMC8799590; doi:10.1007/s10689-020-00224-y)
Supplement: Supplementary file 5 — Quantification of transcripts produced by BRCA2 c.425G>T and controls (DOCX 14 kb) [file 10689_2020_224_MOESM5_ESM.docx]

| **Region Amplified** | **Transcript Observed** | **Control Blood^†^** | **Control Breast Tissue^†^** | **Variant Carrier^†^** | **cDNA** | **Protein** |
| --- | --- | --- | --- | --- | --- | --- |
| Exons 2-8 | Full-length | 100% (97/97) | 96% (91/95) | Pt 1: 46% (59/127)**^‡^**  Pt 2: 55% (90/163)**^‡^**  Pt 3: 45% (71/159)**^‡^** |  |  |
|  | ∆4p | 0 | 3% (3/95) | Pt 1: 0  Pt 2: 0  Pt 3: 0 | c.317_320del | p.Arg107Metfs*13 |
|  | ∆4 | 0 | 0 | Pt 1: 33% (42/127)  Pt 2: 29% (48/163)  Pt 3: 33% (53/159) | c.317_425del | p.Gly106Valfs*10 |
|  | ∆3-4 | 0 | 1% (1/95) | Pt 1: 7% (9/127)  Pt 2: 1% (2/163)  Pt 3: 1% (2/159) | c.68_425del | p.Asp23_Valfs*10 |
|  | ∆3-5 | 0 | 0 | Pt 1: 0  Pt 2: 0  Pt 3: <1% (1/159) | c.68_475del | p.Asp23_Ser158del |
|  | ∆4-5 | 0 | 0 | Pt 1: 11% (14/127)  Pt 2: 13% (22/163)  Pt 3: 16% (26/159) | c.317_475del | p.Gly106_Ser158del |
|  | ∆4-6 | 0 | 0 | Pt 1: <1% (1/127)  Pt 2: <1% (1/163)  Pt 3: <1% (1/159) | c.317_516del | p.Arg107Serfs*9 |
|  | ∆4-7 | 0 | 0 | Pt 1: 2% (2/127)  Pt 2: 0  Pt 3: 3% (5/159) | c.317_631del | p.Gly106_Ile210del |

† % (n isolated traces/total traces)

‡ c.425G only
