# Supplementary material for: Interpretation of BRCA2 Splicing Variants: A Case Series of Challenging Variant Interpretations and the Importance of Functional RNA Analysis
Source: Fam Cancer. 2021 Jan 20;21(1):7–19. doi: 10.1007/s10689-020-00224-y (PMC8799590; doi:10.1007/s10689-020-00224-y)
Supplement: Supplementary file 6 — Quantification of transcripts produced by BRCA2 c.8331+2T>C and controls (DOCX 13 kb) [file 10689_2020_224_MOESM6_ESM.docx]

| **Region Amplified** | **Transcript Observed** | **Control Blood^†^** | **Control Breast Tissue^†^** | **Variant Carrier^‡^** | **cDNA** | **Protein** |
| --- | --- | --- | --- | --- | --- | --- |
| Exons 16-20 | Full-length | 92% (61/66) | 87% (95/109) | 54% (64/119) |  |  |
|  | ∆18 | 0 | 2% (2/109) | 27% (32/119) | c.7977_8331del | p.Tyr2660Phefs*43 |
|  | ∆17-18 | 5% (3/66) | 9% (10/109) | 9% (11/119) | c.7806_8331del | p.Ala2603Phefs*43 |
|  | ∆17q-18 | 0 | 0 | 6% (7/119) | c.7826_8331del | p.Gly2609Aspfs*4 |
|  | Miscellaneous | 3% (2/66) | 2% (2/109) | 4% (5/119) |  |  |
| Exons 14-18 | Full-length | ND | ND | c.7242A: 62% (62/100)  c.7242G: 38% (38/100) |  |  |

† % (n isolated traces/total traces)

‡ Allele of origin: % (n isolated traces/total traces)
